# REDD+ on the rocks? Conflict Over Forest and Politics of Justice in Vietnam

**DOI:** 10.1007/s10745-016-9821-1

**Published:** 2016-04-02

**Authors:** Thomas Sikor, Hoàng Cầm

**Affiliations:** School of International Development, University of East Anglia, Norwich, NR4 7TJ UK; Institute of Cultural Studies, Hanoi, Vietnam

**Keywords:** Forest, Justice, Politics, Reduced emissions from deforestation and forest degradation, Vietnam

## Abstract

In Vietnam, villagers involved in a REDD+ (reduced emissions from deforestation and forest degradation) pilot protect areas with rocks which have barely a tree on them. The apparent paradox indicates how actual practices differ from general ideas about REDD+ due to ongoing conflict over forest, and how contestations over the meaning of justice are a core element in negotiations over REDD+. We explore these politics of justice by examining how the actors involved in the REDD+ pilot negotiate the particular subjects, dimensions, and authority of justice considered relevant, and show how politics of justice are implicit to practical decisions in project implementation. Contestations over the meaning of justice are an important element in the practices and processes constituting REDD+ at global, national and local levels, challenging uniform definitions of forest justice and how forests ought to be managed.

## Introduction

REDD+ (reduced emissions from deforestation and forest degradation) is a global initiative that seeks to mitigate climate change by decreasing losses of the world’s remaining natural forests or other reductions in forest carbon stocks (e.g., Angelsen 2009). At the same time, REDD+ has given rise to significant contestations over the justice of tropical forest management at the global level (for example, how differences in benefits and costs, in influence on decision-making, and in understanding of relevant actors should be handled). Efforts to protect forest people’s rights, needs, and visions are reflected in the REDD+ Safeguards, which provide unprecedented global recognition of indigenous peoples’ rights to forests (Savaresi 2013). The same efforts have led international donors to fund ‘indigenous REDD+ pilots’ for developing approaches to REDD+ that accord indigenous peoples a central role.^1^

Yet, Norway’s Agency for Development Cooperation (NORAD) is funding an indigenous REDD+ pilot in Vietnam where there is barely a tree on the limestone rocks surrounding the 20 villages included in the project. Moreover, the villagers involved in the pilot project include not only ethnic minority Tay, Nung, and Dao people but also majority Viet. The project invites all local households to participate regardless of ethnicity and settlement history. The only significant patch of natural forest left in the area has not been included in the project, although it has long been managed by Dao ethnic minority villagers and, therefore, would neatly fit the purposes of indigenous REDD+.

This paradox, we suggest, contains broader insights about REDD+. Specific REDD+ practices rarely fit general ideas about REDD+ because they develop on top of ongoing struggles and conflict over forests (cf. Mahanty *et al*. 2013). Variation in the practice of REDD+ is not due to aberrations from a grand plan – whether environmental, neoliberal, or neocolonial – but because practices and processes at global, national and local levels constitute a set of negotiated and highly varied arrangements. It is only through specific practices in particular settings that REDD+ is realized, i.e., forest management practices on the ground as much such as negotiations at global summits. Moreover, justice is at the core of negotiations over REDD+, whether international negotiations and national policy debates about equity and governance or local struggles over forest management. Justice concerns are implicit to practical matters commonly encountered in the design and implementation of REDD+ actions.

Here we investigate the practices and processes constituting REDD+ through an examination of a REDD+ pilot implemented by a Vietnamese non-govermental organization (NGO) from 2011 to 2014 in northern Vietnam. We analyze how the implementation of the project affected ongoing negotiations over forests at the site, how various social actors invoked REDD+ in their claims on forests, and how existing conflicts influenced the implementation of the REDD+ pilot. Our particular interest is with a specific element in conflict over forest, which we term *politics of justice* and refers to contestations over the meaning of justice, i.e., the particular notions social actors attribute to justice in a specific context. We are interested less in investigating the effects of REDD+ on differentiated social actors, or the issues of distribution, participation, and recognition raised by its implementation (Leggett and Lovell 2012; Mathur *et al*. 2014), but rather in focusing on social actors’ ideas about what just forest governance is about, how REDD+ would have to be conducted to serve justice, and how these ideas have influenced actual forest management practices. Struggles over the meaning of forest justice, we argue, are a critical element in conflict over forest and the practices and processes constituting REDD+.

We begin with a review of the literature on conflict over forest to develop a simple framework for examining the politics of forest justice organized around three parameters: subjects, dimensions, and authority. We then introduce important background on negotiations over forests in contemporary Vietnam and global struggles over REDD+. In the following section, we analyze negotiations over practical elements of the REDD+ pilot – the location of the project, the selection of the forest to be protected, the designation of eligible participants, the procedures to facilitate their participation, and decisions about what participants receive in return for forest protection – and synthesize the underlying politics of forest justice along the three parameters of subjects, dimensions, and authority. We conclude by developing the implications of our empirical insights for more general understanding of politics of justice and REDD+.

## Conflict Over Forest and Politics of Justice

Justice concerns are an integral part of the normative arrangements underpinning forestry. They commonly inform everyday claims on forests along the lines of Peluso’s (1996) ‘ethics of access’.^2^ Ethical concerns act as ‘motivator of behavior’ (Peluso 1996: 515) in the sense that considerations of intra- and intergenerational justice among others influence forest management practices. They also influence access to and control over forests by lending legitimacy to some forest claims and withholding others (Fortmann 1995). The ethical underpinnings are not fixed in time nor do they apply uniformly within a particular social setting. They can be specific to particular resources (Peluso 1996) and change over time in response to wider political and economic changes. Nonetheless, ethics of access inform claims people make on forests and their forest management practices, thereby mediating the influence of broader economic and political forces (Sowerwine 2011).

Ethical considerations are an important element of environmental politics, whereby the ethical notions applied by various social actors tend to be plural. In Peluso’s (1996) case, villagers developed different ‘ethics of access’ to durian, other fruit trees, and rubber. Moreover, villagers’ notions differed from those informing state governance interventions such as privatization. In environmental justice scholarship, it is recognized that multiple notions of justices are found amongst diverse actors (Sikor 2013a; Walker 2011; see also He and Sikor 2015; Martin *et al*. 2014).

Consequently, conflict over forests may arise because social actors disagree on applicable notions of justice. As people disagree on the meaning of justice, they may contest justice along three key parameters: subjects, dimensions, and authority (cf. Fraser 2009). First, claims on forest may invoke different *subjects of justice*, i.e., which social actors are considered to legitimately possess rights and bear responsibilities relating to forest. For example, Peluso (1996) explains how both lineages and individuals (as different subjects) possess certain rights to forests: inheritance rights to durian and other fruit trees are shared within lineages, while individuals acquire rights to fruits through labor investment. Bose (2013) describes the tension between the Indian Forest Rights Act, which calls for the formalization of customary rights on a household basis, and tribal people’s assertion of collective forest rights on the basis of their collective identity. Elmhirst (2011) notes how negotiations over access to forests in Indonesia simultaneously involve contestations over subjects, since women are granted access through membership in a household rather than as individual citizens.

Second, forest claims may assert different *dimensions of justice*, which can be broadly distinguished as redistribution, representation, and recognition (Schlosberg 2004; Walker 2011), and some dimensions of justice may be more valued than others in given socio-political contexts or by certain actors. Different dimensions of justice may explain why villagers may resist co-management initiatives, even where those deliver tangible benefits: aspects of participation and recognition may be more important to them than redistributive actions (Edmunds and Wollenberg 2003). Similarly, the Indian government’s redistribution of individual tenure rights to tribal households encounters opposition because it does not meet their demands for recognition of traditional kinship relations and connectedness to ancestral land (Bose 2013). Benefit-sharing arrangements in Uganda intended to distribute benefits from forests fail to respond to indigenous Batwa demands for recognition of historical experiences of economic and cultural marginalization (Martin *et al*. 2015).

Third, conflict over forest may be due to claims invoking competing institutions of *authority to realize justice* (cf. Sikor and Lund 2009). Forest claims require authorization by institutions to be recognized as legitimate. For example, negotiations over forests in Albania and Romania call upon local governments, central government agencies and various customary institutions to sanction rights (Sikor *et al*. 2009). In Vietnam, conflict over forest is not simply due to discrepancy between state and customary rules but also because villagers justify their claims in reference to both state and customary rules, thereby contributing to the constitution of state and customary authority over forest (Sikor 2011b). More broadly, the devolution of forest tenure rights to local communities opens up grounds for new contestations over what kinds and rules of authority should apply (Larson *et al*. 2010).

Thus, contestations over the meaning of justice involving the three parameters of subjects, dimensions and authority are a key element in conflict over forest. Conflict arises as a result of politics of justice originating from multiple notions of justice.

## Forest Politics in Vietnam and Global Struggles Over REDD+

### Negotiations Over Forests in Contemporary Vietnam

The people living in and around forests in Vietnam come from a wide range of cultural and historical backgrounds including majority Viet as well as 53 state-classified ethnic minorities. Ethnic minorities account for 14 % of the national population and are largely concentrated in the uplands, where most of the country’s forests are located (Sikor 2011a). They are generally considered to have lived longer in the uplands than the Viet, who started to migrate there in larger numbers in the 1950s (Hardy 2003).

Vietnam’s constitution grants equal citizenship rights to all people regardless of their ethnicity, while recognizing ethnicity as an individual attribute. After gaining independence in 1954, the Vietnamese Communist Party quickly abandoned a previous policy based on the Soviet policy of recognizing the right of self-determination for some peoples and instead sought to unify all ethnic groups under the premise that the ethnic minorities share a common country with the Viet, and has ruled out any recognition of ethnic peoples’ collective identities as indigenous peoples (Keyes 2002). In the constitution, members of all ethnic groups are considered citizens with equal socio-political and cultural rights.

Perhaps the most important state policy affecting the relationship between people and forests in Vietnam over the past three decades was forestland allocation, which granted households the right to use previously state-owned forestland for 50 years irrespective of their ethnicity. By the end of 2013, households possessed nearly a quarter of the country’s total forestland area (Kiem 2014). In contrast, village communities had received use rights to only 3 %.

Beyond forestland allocation, Vietnam’s forest policy has a strong focus on distributive matters. With no significant governance reforms, most attention has been on the implementation of large reforestation and forest protection programs. The 327 Program in the 1990s and 661 Program in the first decade of the 2000s used large-scale central government financial support to households throughout the country for forest protection and tree plantations. This emphasis also characterizes the most recent policy initiative known as Payments for Forest Ecosystem Services (To *et al*. 2012).

Forestland allocation has not put an end to nationwide disputes over forests due to the discrepancy between state regulations and customary use and management, which underlies the persistence of logging deemed illegal by state rules but often considered legal by villagers (McElwee 2004). Villagers across the country have exploited timber from surrounding forests even though they are under state protection regulations (Cam 2011). Similarly, village communities often manage areas that government statistics call forestland, bare land, wasteland or unused land, for crop cultivation and animal husbandry (McElwee 2011). Forestland allocation may even have intensified conflict over forest because it has often implied a massive re-assertion of state control over land (Sikor 2011b).

Thus, Vietnamese state policy has been guided by the centrality of the household as the subject of justice and distributive matters, and the state as the sole politico-legal administrator of justice, described here as ‘politics of possession’ tying household-based citizenship to state provision of economic entitlements (see Sikor 2012). Yet these ideas continue to lead to disputes over forests in many parts of the country that are not only about what politico-legal institutions should exercise authority over forests but also challenge notions of citizenship based in households and the focus on distribution as the most critical dimension of justice.

### Global Struggles Over REDD+

Indigenous peoples’ demands for self-determination have been a central tenet of global struggles over REDD+. After the 2007 conference endorsed preparatory work on REDD+ under the United Nations Framework Convention on Climate Change (UNFCCC), and intense lobbying by indigenous peoples and their supporters to include references to indigenous peoples’ rights in UNFCCC decisions (Schroeder 2010), UNFCCC Decision 1/CP.16 identified ‘indigenous peoples,’ ‘local communities,’ and ‘members of local communities’ as subjects of justice next to the traditional concern with nation states in global agreements -- an unprecedented recognition of indigenous peoples’ and local communities’ collective identities and rights in global forest regulations (Savaresi 2013; Walbott 2014).

Another critical issue in global contestations over REDD+ has been controversy over the dimensions of justice to be regulated in UNFCCC decisions. Indigenous and forest rights activists demanded the inclusion of text speaking to the urgency of redistribution, such as the need for forest tenure reform to formally recognize the customary use and management of forests and the importance of equitable sharing of REDD+ benefits and burdens (Luttrell *et al*. 2013; Sunderlin *et al*. 2014). However, text addressing distributive matters at the local or national levels is absent from the Cancun Agreements, even though REDD+ at the global level is primarily conceived as a distributive mechanism (Sikor 2013b). Instead, UNFCCC Decision 1/CP.16 defines participation and recognition as the relevant dimensions of justice. The so-called fourth REDD+ safeguard states the necessity of involving relevant stakeholders in the design, development, and implementation of REDD+ actions. Together with the references to ‘indigenous peoples’ and ‘local communities,’ the third REDD+ safeguard provides certain recognition to forest people’s historical experiences and particular stake in forests, acknowledging their ‘knowledge and rights’ and alluding to the 2007 United Nations Declaration on the Rights of Indigenous Peoples (UNDRIP) (Walbott 2014).

The attention to indigenous peoples’ and local communities’ rights has implications for the recognition of authority beyond the nation-state, which has been another key demand by international forest right activists. Drawing on UNDRIP, activists demanded to make the principle of Free Prior and Informed Consent (FPIC) a prerequisite for the design and implementation of REDD+ actions, which would have obliged governments, international organizations, and the private sector to consult and cooperate with potentially affected peoples and communities *through their own representative institutions* (Savaresi 2013). Nevertheless, the UNFCCC decision eventually did not depart from the traditional focus on nation states. States represented through their governments are the key designers and implementers of REDD+ programs and are in charge of implementing and reporting on the REDD+ safeguards.

While UNFCCC decisions recognize forest peoples’ collective identities, the Vietnamese state continues to emphasize equal individual rights to forest and land regardless of ethnicity. The focus on participation and recognition in the REDD+ safeguards is juxtaposed with an emphasis on distributive matters in Vietnam. The conceptions of authority enshrined in UNFCCC decisions and Vietnam’s constitution overlap in their sole focus on the state, yet they encounter resistance both globally and within Vietnam. The REDD+ pilot project we examine here brought a lot of these elements together: it was implemented by a Vietnamese NGO, which we call Verda funded by NORAD, and supported by the international advocacy group for indigenous rights, Tebtebba.^3^

## Negotiations Over Forest on the Ground: The REDD+ Pilot

Verda began implementation of the REDD+ pilot project in the middle of 2010. This involved identification of a site, including a particular forest to be targeted for protection, deciding what villagers to involve, development of a protocol for villagers’ participation, and consideration of what reward the villagers would get in return for their participation.

### Identifying a Project Site: Global, State and Customary Rules

The NGO initially selected a commune that includes rich natural forest and is inhabited by a large majority of ethnic minority Tay people, in line with global REDD+ regulations. However, 6 months into implementation, the provincial People’s Committee decreed a stop to the project at the initial site because it was within an area designated as national security zone, which prohibited the presence of any project with international involvement. In addition, the commune’s forest belonged to a nature reserve, which meant that it was under the management of a national-level protection board and thus outside the local government’s mandate.

Verda consequently selected Linh Bong, a commune in the same district, as the new implementation site, expecting to avoid encountering similar bureaucratic barriers if they targeted the limestone hills designated as forestland around the commune’s 20 villages. As Chi Bach, the local project coordinator, explained in 2014: ‘Most of the forestlands are rocky hills without owners and are managed by the Commune People Committee so that the implementation would be more convenient.’ Since the forest located on the hills had not been assigned to individual households under Vietnam’s forestland allocation program, Verda assumed that it was ‘without owners’ and would benefit from the introduction of suitable management interventions. And because the hills were under the formal management of the Commune People Committee, no higher-level government units held a direct mandate over the hills.

Verda was not concerned that much of the limestone hills was bare of any forest, i.e., there was not much potential for future deforestation or forest degradation that could be reduced: most of the trees that had covered the hills had been cut down by villagers and other loggers during the 1990s. Consequently, the villagers no longer attributed much economic significance to area because no trees of commercial value remained. As a villager observed with regret: ‘It would have been better if the project were implemented here about 10 years ago, since at that time there were still a lot of big trees in the forests’.

Despite awareness of the discrepancy with global REDD+ rules Verda included all the forestland located on limestone hills in the REDD+ pilot. Having commissioned forest inventories, they deemed about half of the forest (620 out of 1168 ha) as potentially suitable for REDD+ since they expected natural forest to regenerate there. However, after consultation with the Commune People’s Committee, Verda decided to include all the forestland on limestone hills in the project. Chi Bach explained that they wanted to contribute to general forest protection, as was common practice in Vietnam’s large-scale forest protection programs.

The focus on the rocks helped to ensure that forest protection did not cause negative effects on villagers’ livelihoods and customary land use, since the villagers used other forestland located on gentle slopes for crop cultivation, and considered that they have established possession of the land on a customary basis since the 1950s, even though they have received land titles to only a minor share of the land. The importance of respecting customary rules was not lost on a commune cadre who commented: ‘We respect the history of land use practices as all non-rocky mountain forests are local people’s production forests.’

Thus, Verda’s search for a suitable project site involved intense negotiations with government officials and villagers. The underlying question was about which rules should apply to the designation of the project site: UNFCCC regulations, the land use classifications and territorial zoning done by the Vietnamese state, or customary rules practiced by villagers. Verda initially found a site that met the rules of UNFCCC and NORAD, as it had rich forest and a large majority of ethnic minority Tay residents. Yet due to state regulations, they had to move to a site with very little forest on rocky hills (and a more complex ethnic mix – see below). Moreover, they ended up including much forestland that they did not deem suitable for REDD+ under the UNFCCC, while at the same time, professing a strong commitment to respect villagers’ customary use of land in line with the global REDD+ safeguards.

### Determining the Forest to be Protected: Conflict Between Customary Uses and State Designations

When Verda moved the REDD+ pilot to Linh Bong it hoped to include some 400 ha of dense natural forest located at the periphery of the villages. However, on their first visit to the commune it became clear that the forest was the subject of dispute between Dao villagers of Linh Bong and a private forest company from the neighboring province. The Dao of Ba Don and Bu Don villages claimed customary rights to the forest, but the company had obtained a land title from the People’s Committee of the neighboring province after the forest had been transferred to its territory.

The Dao asserted customary rights to the land because they were the first to arrive to the area and had cultivated the land for many years. As an elderly man of Ba Don village commented, ‘When I was a child, there were only Dao living in this area. Today, there are about 12 Nung and 10 Viet families but they are all new migrants. The Viet and the Nung of this village do not have forests because they are newcomers.’ The Dao did not consider that their rights extended to later settlers in the villages.

Until recently, the Dao had considered the land in dispute to be their own. Their fields extended all the way to the border with the neighboring province, which had followed a watershed since the first half of the twentieth century. The same elderly Dao man explained, ‘Land at the far side of the hill belongs to [the neighboring province], and this side belongs to [our province].’ However, in the late 1990s much of land used by the Dao was transferred to the neighboring province under a nation-wide territorial planning project. Once the transfer took effect, the People’s Committee of the neighboring province granted the land to a private forest company. Nonetheless, the Dao were able to continue their customary use of the forest another 10 years. The dispute erupted only after the People’s Committee issued a land title to the company in 2011, and the company constructed a new feeder road into the area, banned any further use of the land, logged over some of the forest, and started to plant trees.

The Dao villagers resisted their exclusion from the land by all possible means. They initially wrote several petitions to the Commune People’s Committee. When they got no a response, they send another, this time to the District People’s Committee, which caused the Commune People’s Committee to criticize them for going over their heads. Frustrated with the lack of responsiveness to their concerns, the Dao took to violence in February 2012. About 70 Dao women and men went to the land under dispute, beat up a few company workers, and destroyed the company’s house and office equipment. The violence caused the police of the neighboring province to arrest four Dao men and a Dao woman, including the head of Bu Don village, keeping them in jail 4 months and forcing them to pay some US$2800 to the company in compensation. In addition, a cadre from Linh Bong’s People’s Committee came to the villages to confiscate land titles that the Dao villagers had received for the disputed land in the 1990s.

The Dao’s appeal for help to Verda did not yield a response. Some of them raised the issue in a meeting with Verda attended by its national director, reasoning that ‘Verda [was] encouraging the local people to protect forests while the [company] [was] destroying our watershed forests.’ They suggested that ‘it [was] not appropriate for Verda to not do anything to stop them,’ considering the project was designed to protect forest. But Verda did not include the land under dispute in the REDD+ pilot even though it had dense natural forest, protection of which was the stated target of global REDD+ regulations. Chi Bach, the coordinator, argued that they could not include the forest because ‘we implement our pilot projects in land areas with clear boundaries, not in disputed forests.“ Verda could become active only after the dispute was resolved.

The Company submitted their application for the land title to the provincial People’s Committee after Verda had started work in Linh Bong commune. Interviewed in April 2014, its director said that they knew about ‘the project implemented by international organizations’ in Linh Bong when they submitted their application. He also mentioned that they were hoping to “receive benefits from REDD+ in the future”. To villagers, the connection between the REDD+ pilot and the Company’s actions was clear, as expressed by a Dao men from Bu Don:

‘The arrival of the REDD+ project here with a plan to include all natural forests of the village into the project caused the company to go there to keep the land […] the company is afraid of losing both the natural forests and swidden lands, so they went before us to take the land, which in turn led to the conflict.’

Thus, Verda decided to ignore the dispute. It had to engage in intense negotiations with villagers, government officials, and the private company about which rules applied to the implementation of the REDD+ pilot. The Dao of Ba Don and Bu Don wanted recognition of their customary rights to the forest. The local government upheld the administrative boundaries set by the state through territorial zoning. The company made specific moves to assert its control over the land and establish a claim on future REDD+ benefits on the basis of state law. Verda’s primary concern was getting REDD + -like actions implemented, i.e., to designate forestland for protection, measure forest parcels and conduct forest inventories, even if the forest that was of highest importance according to UNFCCC rules was not included, and an opportunity to address global concerns about indigenous peoples’ customary rights was missed.

### Deciding on Participants: Global Definitions, National Citizenship, and Local Identities

The third issue Verda had to resolve was about which villagers from Linh Bong should be targeted through the REDD+ pilot and were eligible to participate.

Verda’s application submitted to NORAD stated that the REDD+ pilot was designed to build the capacity of ethnic minority communities to participate in REDD+ actions. Ethnic minority people chosen to represent the indigenous peoples flagged in global REDD+ regulations as deserving special treatment and to whom REDD+ pilots should be targeted. Consequently, Verda initially chose a commune for project implementation where ethnic minority Tay people accounted for almost the entire population. When it was forced to move the project, one of the reasons that Linh Bong was chosen was the presence of ethnic minorities.

However, Linh Bong’s settlement history and ethnic composition turned out to be complex. The commune’s population of 5900 people belongs to a total of seven ethnic groups, including large numbers of Nung, Tay, and Dao, as well as a few Hmong, Cao Lan, and San Chi households. However, majority Viet is the largest group (49 %) and their ancestors had arrived in the area first - some families claim their ancestors came 17 generations ago. However, not all Viet families claim descent from the first settlers. Others arrived as late as 1962–63 from the Red River Delta under a state resettlement program. The first Dao families had come to Linh Bong seven generations ago, followed by Nung and Tay families five generations ago. Many Nung and Tay had settled only in the 1960s, 1970s, and 1980s, when they joined the existing residents in collective wet-rice cultivation and established their own upland fields around the villages.

Viet and Dao villagers took significant pride in the fact that they had opened up the area for settlement and cultivation. For example, a Dao elder recalled:

‘When I was a child, Bu Don had only seven families. At that time, there were no ethnic [Tay] and Bu Don was covered by very dense forest. I went to the forest to find land to do shifting cultivation while my parents cultivated paddy fields at the foothills. The paddy field that my parents cultivated had originally been developed by my grandparents, who passed them on to my parents.’

When Linh Bong’s agricultural cooperatives divided up the collective wet-rice fields under the general trend of decollectivization around 1990, it was the villages’ settlement history that was considered, but not ethnicity per se. The villagers decided to restore wet-rice fields to their historical owners and heirs in contrast to the state policy of distributing agricultural fields equally among the current workforce. The villagers’ decision meant that the Viet, Dao, Nung, and Tay families who had settled in Linh Bong and established wet-rice fields before 1960 received their fields back. Those Viet, Dao, who had moved to Linh Bong from the 1960s onward had joined the agricultural cooperatives after their arrival and had never worked their own wet-rice fields.

Verda thus found itself in a delicate situation with regard to the question of who was eligible for participation in the REDD+ pilot. The project proposal’s focus on ethnic minorities did not match with local settlement history. More importantly, villagers did not see any sense in distinguishing local residents by their ethnicity. They commonly mentioned that all commune residents had a migration background, though some had arrived earlier than others. In matters of production, they asserted that all of them enjoyed equal rights. As a villager commented, ‘both Viet and other ethnic groups have lived in the region together for quite a long time. [… the project] could not be implemented successfully without participation of Viet.’ Verda resolved the dilemma by inviting all households in the commune to join the REDD+ pilot, thereafter referring to ‘ethnic minorities and communities living in and around forest’ as the project’s target group. As Chi Bach explained, ‘it is true that this project is designed for poor ethnic minority people, yet it could not be done without inclusion of the Viet.’ This decision to include villagers from all ethnic groups, whether minority or majority, matches with the UNFCCC decision to identify ‘indigenous people’, ‘local communities’ and ‘members of local communities’ as subjects of justice.

### Developing a Protocol for Villagers’ Participation: The Cooperatives

Once Verda had decided who would be eligible to participate, it had to develop a specific protocol for participation. International activists and UNDRIP were clear about a suitable means to enable indigenous peoples’ participation in REDD+: consultation following the principle of FPIC. Before any REDD+ action was designed or implemented, potentially affected indigenous peoples should be consulted through their own representative organizations. The emphasis on FPIC, therefore, was on collective representation, as reflected in the focus on ethnic minority *communities* in Verda’s project application.

With approval by the Commune People’s Committee, Verda founded two ‘agricultural, forestry and environment cooperatives,’ each having a chairman and a management board including the village heads and the leaders of the cooperative’s production units. Altogether, the cooperatives were made up of a total of 60 self-management teams including about 25 households each.

Membership in the cooperatives was voluntary. Any villager who wanted to join was invited to submit an application for household membership, to be signed by both the husband and wife. The head of the self-management team and the cooperative management board reviewed the application for approval. Membership required households to ‘comply fully with all regulations and rules of the cooperative and the self-management team as well as Verda’s requirements.’ Additionally, if households did not want to join the cooperatives, they were obliged to sign a formal pledge that they forewent any claims on future benefits disbursed by the cooperatives but would comply with ‘the regulations [of the cooperative] on forest use and management under the REDD+ demonstration project.’

According to Chi Bach, this procedure satisfied the demands of FPIC. She did not mind that the cooperatives were generally considered economic organizations and not consultative mechanisms, and saw no discrepancy between the emphasis on collective consultation under FPIC and Verda’s invitation to villagers to join the cooperatives on a household basis. Similarly, she had no problem with giving villagers a single choice about whether to join or not to join the cooperatives rather than consulting them more generally about various options for forest protection.

The cooperative model was in line with government policy and provoked no disapproval from villagers. Nevertheless, it differed significantly from the globally agreed options for facilitating people’s participation in REDD+ projects, particularly the FPIC principle. In Linh Bong, villagers had the choice of opting in or out of activities determined and decided by Verda in consultation with the Commune People’s Committee.

### Rewarding Villagers: Agricultural Extension

The fifth issue that Verda had to address was about the nature of rewards to villagers in return for forest protection. The potential rewards ranged from cash payments and the provision of material assistance to activities that would enhance villagers’ role in forest governance or support the recognition of the immaterial values that they might attribute to the forest. As noted, the global REDD+ Safeguards emphasize efforts to increase people’s participation in forest governance, recognizing their knowledge, and protecting their rights.

When Verda assigned specific parcels of forestland to the self-management teams for protection, it sought to make sure that villagers would also have rights to the land and the benefits derived from it. Verda arranged for the measurement of all forest parcels by technical experts and their mapping in collaboration with villagers. It distributed the forestland to all self-management teams in parcels of 25 ha each, demarcated the forest parcels in the field, and put up signs banning any further logging. On its request, the District People’s Committee issued a decision that granted the self-management teams tenure rights to the parcels for a period of 50 years. However, Verda did not request the People’s Committee to issue legal land titles to villagers, as those would have required complex bureaucratic procedures involving various government units.

Even though the forest parcels possessed little material value and no land titles were issued, their assignment to the self-management teams caused significant debate among the villagers. Villagers initially wanted to distribute forest parcels among teams on the basis of geographical proximity, a proposal that Verda and the Commune People’s Committee approved. Nevertheless, the allocation principle remained subject to ongoing debate at several cooperative meetings. Moreover, after villagers inspected the forest parcels in question and discovered that the forest was of better quality in the more remote locations, many changed their demands and now wanted to be allocated parcels in remote areas. The issue was finally resolved when Verda promised that future revenues from REDD+ would be distributed equally among all self-management teams independent of the actual carbon contained in their respective forest parcels. In Chi Bach’s words, the revenues would be ‘shared between people managing rocky hills *with* forest and people managing rocky hills *without* forest.’

In addition, Verda provided various kinds of support to local agriculture in reward of villagers’ involvement in forest protection. Helped by their director’s background as an agricultural expert, Verda implemented various ‘livelihood improvement activities’ promoting the cultivation of commercial crops although there was no direct link between those and the protection of the degraded forest on the rocky hills. They used the cooperatives to supply seed and fertilizer to villagers for the cultivation of various cash crops and contracted extension experts to train villagers in improved cultivation techniques. They also granted the two cooperatives a loan of US$28,000 to generate immediate material benefits to villagers and thereby encourage their involvement in the cooperatives. Consequently, the REDD+ pilot came known to many villagers as an ‘agricultural extension project.’

Distributive concerns were thus at the core of discussions about the implementation of the REDD+ pilot in Linh Bong. All involved actors – Verda, the Commune People’s Committee, and villagers – emphasized issues of distribution with regard to what villagers received in return for their involvement in forest protection. Since the forest had no significant material value, agriculture became the focus for the provision of material benefits.

### Politics of Justice in Linh Bong

The negotiations over forest we have described were not simply about the practical conduct of the REDD+ pilot but involved contestations over the meaning of forest justice. The questions Verda, villagers, and the local government dealt with about where the project should be implemented, what areas should be targeted, who should participate, how people should participate, and what villagers should get in return for forest protection, all touched on matters of justice. The contestations were centered on specific issues of justice, such as the dispute between the Dao and the company and the question how forest parcels should be distributed among self-management teams. Simultaneously, they addressed the core parameters of justice: subjects, dimensions, and authority.

Different ideas about *subjects of justice* underlay the debates about who should participate in the REDD+ pilot, and how participants should be involved. Verda’s application to NORAD struck a compromise between global REDD+ politics and Vietnamese law, highlighting a priority concern with ethnic minority communities but avoiding any further implications of indigenous peoples’ agenda. Ethnic minority communities took the place that indigenous peoples had held in a previous international collaboration, which had involved Verda as the national partner and was led by the international advocacy group Tebtebba. In that project, Verda had stated its goal as ‘ensuring the effective participation of *indigenous peoples* in global and national REDD processes’ [emphasis added].^4^ In the new project, it replaced the term ‘indigenous peoples’ with ‘ethnic minorities,’ a common practice in Vietnam. At the same time, Verda retained the emphasis on collective identities by speaking of ethnic minority communities.

When the project was implemented, villagers, Verda and the local government agreed to invite all households in Linh Bong to participate, and Verda quickly modified its language to refer to ‘ethnic minorities *and communities* living in and around forest’ (emphasis added). In practice, project activities engaged participants as individual households, which reflected a general consensus in Linh Bong that ethnicity did not differentiate villagers’ rights to land and forest. Instead, villagers’ rights depended on the labor that households had invested in land. Because no household had invested any labor in the forestland on the rocks, no one could claim any preferential rights to the forestland included in the REDD+ pilot.

Similarly, there was not much debate about the applicable *dimensions of justice* in Linh Bong. From the outset, Verda, villagers and the local government shared the premise that villagers deserved material rewards in return for forest protection. Their focus was squarely on the distributive dimension of justice in the form of agricultural support and tenure rights to forestland.

For most villagers, this was a question of how much agricultural support Verda would deliver to them in return for forest protection, and how the support was distributed among households. They did not mind that Verda set up economic cooperatives to organize interactions with villagers, and dictated the rules governing them. They appreciated Verda’s efforts to strengthen their tenure rights to the rocky hills, and engaged in a prolonged discussion about how parcels should be distributed among the self-management teams. For the affected Dao, rights to forestland were a more serious matter, as demonstrated by their resort to violence in their struggle against the company. Ultimately, they were the only people in Linh Bong who felt negatively impacted by the REDD+ pilot since their distributive demands found no support from the local government or Verda.

In terms of *authority*, Verda constantly had to decide whose rules applied to their project – those of the UNFCCC, Vietnamese law and policy, or customary practice. It had to move the project away from the original site to Linh Bong due to Vietnamese national security concerns. In Linh Bong, it avoided targeting forestland on the gentler slopes where villagers had effectively established customary claims to its use for crop cultivation. At the same time, Verda did not support Dao customary claims to forest against state delineations and the company. As a result, the REDD+ pilot ended up protecting highly degraded forestland and inviting all households to get involved.

While broader authority relations remained contested in Linh Bong, the state became the primary politico-legal institution of distributive justice for households. The District People’s Committee issued the decision to grant self-management teams tenure rights to the rocky hills. It was also state law and regulations that led Verda to set up the cooperatives for organizing forest protection and assisting agriculture. And it was with reference to state law that it refused to lend support to the Dao villagers’ customary claims. Verda’s REDD+ pilot thus served the consolidation and extension of state authority in Linh Bong.

## Conclusion: Politics of Justice and REDD+

Justice is a key element in negotiations over REDD+ practices as they develop in situations of ongoing conflict over forest, not only in terms of the distributive, procedural and recognition issues raised by REDD+, but also because the various stakeholders attribute different meanings to justice in specific contexts. The contestations involve competing ideas about the subjects of justice, the dimensions of justice considered relevant, and authority realizing justice. They influence the arrangements recognized as REDD+ and their prospects to contribute to climate change mitigation and just forest governance.

Similar to what we observed in the REDD+ pilot in Vietnam, we surmise that REDD+ projects witness politics of justice in other places, on the ground as well as in debates at the national and global levels. The actors involved in REDD+ actions are likely to disagree on the meaning of justice along the key parameters of subjects, dimensions, and authority. Politics of justice may play a role in policy debates about the equitable distribution of REDD+ finance and responsibilities among countries (Di Gregorio *et al*. 2014), the recognition of indigenous peoples at the global level (Savaresi 2013), or forest tenure reform as a vehicle to redistribute forest rights or recognize customary authority (Mahanty *et al*. 2013). Contestations over the meaning of justice also underlie the concerns of REDD+ practitioners over benefit distribution systems (Luttrell *et al*. 2013). Yet, politics of justice may more often remain implicit to practical matters of REDD+ design and implementation, such as the measurement of carbon stocks (Sikor 2013b).

Contestations over the meaning of justice influence the governance arrangements known as REDD+ in ways similar to struggles over knowledge and technology. The meanings and implementation of justice are critical elements of the relations between those seeking to govern and those whose conduct is to be governed. It is possible that some meanings may become dominant, assuming a force equivalent to the construction of certain knowledge truths about forests (Forsyth and Walker 2008) and conservation (Carrier and West 2009). Yet the meaning of forest justice may also remain contested, resulting in a perplexing variety of actual REDD+ practices, such as rewarding villagers for the protection of rocks in Vietnam.

Contestations over justice pose a radical challenge to global efforts of defining REDD+ in terms of certain technologies and practices. The multi-sited nature of contestations over the meaning of justice challenges the simplifications required by global-level attempts to institute uniform regulations for forest-based climate change mitigation (Mahanty and McDermott 2013). Global perspectives on the needs, interests and rights of indigenous peoples may not match the claims and notions of forest justice asserted by marginalized people at the local or national level (Li 2002). Ideas about self-determination, local governance, and customary authority may not have much traction in many contexts with disempowered people, who view the nation state as a more desirable politico-legal institution for realizing justice (Ribot *et al*. 2008). Emphasis on procedural justice and recognition, as reflected in the importance attributed to FPIC by indigenous advocacy groups, may not meet the expectations of marginalized people who prioritize distributive matters (cf. Upton 2014).

Nevertheless, global regulation, in particular the REDD+ safeguards, has potential to serve forest justice. REDD+ may develop into a platform that helps to empower marginalized people by asserting universal goals if safeguard processes simultaneously provide sufficient space for adaptation to marginalized people’s specific ideas about justice (Forsyth and Sikor 2013). However, one can also envision other scenarios of how global safeguards will not help just causes. They may not help to bring about justice simply because they may not develop enough traction on the ground due to the overpowering influence of local and national definitions of justice. At the same time, global safeguards may even work to undermine efforts to make forest governance more just if they impose rigid categories on national and local politics of justice, or if national and local implementers employ top-down approaches to operationalize forest justice.

Overall, REDD+ may witness Vietnamese villagers protecting rocks, but it may not be on the rocks yet. The politics of justice highlighted in this paper may look like a constraint on the implementation of global ideas and rules about REDD+ from a narrowly technocratic perspective. However, a switch of perspective may help REDD+ designers and implementers to recognize the potentials of engaging with justice concerns actively (cf. Gritten *et al*. 2009). Ideas of justice have always been integral to forestry, assuming a central place in everyday negotiations over forest practices and constituting a central tenet of professional forestry. Renewed attention to justice, particularly differences in ideas about justice, may help policy-makers, professionals, activists and villagers to recognize the potential of justice concerns as motivation for sustainable forest management. Thus, REDD+ may provide a new opportunity to take a fresh look at not only forest justice but also more broadly at sustainable forest management.

## Footnotes

1 For example, NORAD has funded a number of pilot projects on REDD+; see http://www.norad.no/en/front/funding/climate-and-forest-initiative-support-scheme/grants-2009-2012

2 Arguably, this also applies to state forestry and the forestry profession, for which priority on ‘the common good’ and national development have been foundational (Peluso 1992).

3 We use pseudonyms for all persons, places, and organizations in Vietnam to protect the anonymity of our informants.

4 See http://www.indigenousclimate.org/index.php?option=com_content&view=article&id=92:cerda&catid=37:our-partners&Itemid=84, accessed 16/09/2014
